# Distinct differences in analytical performance of two commercially available assays for specific IgE to egg white and house dust mite allergens

**DOI:** 10.1186/s12948-021-00151-y

**Published:** 2021-08-02

**Authors:** Komei Ito, Kazunori Tagami

**Affiliations:** 1 Allergy and Immunology Center, Aichi Children’s Health and Medical Center, Obu, Aichi Japan; 2 Kasugai Municipal Hospital, Kasugai, Aichi Japan

**Keywords:** Allergy, Specific IgE, ImmunoCAP, Immulite, Chimeric Antibodies, Comparison

## Abstract

**Background:**

Measurements of allergen-specific IgE antibodies with different manufacturers’ assays show modest or poor agreement. This study compares analytical performance of specific IgE tests for whole allergen extracts and individual allergen components of two assay systems, IMMULITE and ImmunoCAP, using human sera as well as monoclonal antibodies.

**Methods:**

Comparisons were performed for specific IgE to house dust mite (HDM, n = 44), egg white (EW, n = 36) and the allergen components rDer p 1, rDer p 2, nGal d 1, nGal d 2 and nGal d 4 in human sera and with monoclonal mouse/human chimeric IgE antibodies specific for the same allergen components. Competitive interference with IgE measurement was investigated using allergen-specific monoclonal IgG and IgG4 antibodies.

**Results:**

Measurements of IgE to HDM and EW in serial dilutions of human sera revealed weaker dilution linearity with IMMULITE than with ImmunoCAP. Analysis of five different monoclonal IgE antibodies with total and specific IgE assays, expected to return similar levels, gave an average specific/total IgE ratio of 0.96 (range 0.71–1.14) with ImmunoCAP and 1.89 (range 0.76–2.85) with IMMULITE, indicating overestimation of specific IgE by IMMULITE. With the EW IgE tests of both assay systems, measurements of a chimeric anti-Gal d 2 IgE antibody were unaffected by a competing mouse IgG antibody. While the same was true for measurement of a chimeric anti-Der p 1 IgE antibody using the HDM test in ImmunoCAP, a suppression of measured concentrations by up to 42% was observed in IMMULITE. Similarly, measurement of HDM-specific IgE in human sera by ImmunoCAP was unaffected by a competing monoclonal anti-Der p 2 IgG4 antibody while IMMULITE displayed a reduction of HDM-specific IgE values by up to 30%.

**Conclusions:**

In this evaluation of analytical performance of two widely used assay systems, ImmunoCAP showed higher accuracy in quantitation of specific IgE and greater resistance against competing allergen-specific non-IgE antibodies which may arise through natural or dietary exposure, or as a result of allergen immunotherapy treatment.

## Background

House dust mite (HDM) allergy has a significant clinical effect on lung function in children [1]. Among patients with asthma and/or rhinitis, a prevalence of HDM sensitization of 22% in Europe, around 35% in United States and up to 80% in Asia has been reported [2–4]. Hence, proper management of HDM allergy is of outmost importance and may significantly attenuate the development of asthma and/or rhinitis. A conclusive diagnosis of HDM allergy in patients with asthma and/or rhinoconjunctivits can be reached through a carefully taken medical history in combination with specific immunoglobulin E (sIgE) testing. If a patient is suspected of being HDM allergic, it is of importance to investigate mite exposure at the patient's residence [5]. In addition to whole HDM extract, there are several major HDM allergen components commercially available for IgE testing, e.g. Der p 1, Der p 2 and Der p 23 [5–7].

As far as food allergy is concerned, egg white is one of the most common causes in children. Prevalence figures of 0.5–2.5% have been reported [8] and its diagnosis is based on clinical history, specific IgE tests and standardized food challenges [9]. Quantitative determination of egg white (EW)-specific IgE enables prediction of allergic reactions in oral food challenge (OFC) testing as well as monitoring of oral immunotherapy. Probability curves providing the likelihood of a positive outcome of an OFC or of manifest symptoms to egg have been established, based on levels of specific IgE to both EW and Gal d 1 (ovomucoid) [10–12].

Allergic reactions can occur either to any form of egg or, in some patients, mainly to raw or lightly cooked / baked egg [13, 14]. Clinically, Gal d 1 specific IgE antibody levels can be used to predict reactivity or tolerance to hard-boiled or baked eggs. For this purpose, positive and negative decision points levels for Gal d 1-specific IgE have been reported. In Palosuo et al. [15], all subjects with levels greater than 14 kU_A_/L (ImmunoCAP) failed an egg OFC while 95% of those with a level less than 0.9 kU_A_/L passed an egg OFC. Similar results were reported by Ando et al. [16], defining decision point levels of 10.8 kU_A_/L and 1.6 kU_A_/L, respectively. Thus, when measuring Gal d 1-specific IgE antibodies, high test precision is clinically important, not only in the high concentration region but also at low concentrations. In addition to Gal d 1, there are other important EW components such as ovalbumin (Gal d 2) and lysozyme (Gal d 4).

It is of utmost importance in sIgE testing that measurements are truly specific and accurate. Currently, there are three commercially available specific IgE immunoassay platforms in Japan, of which IMMULITE® 2000 (Siemens Healthcare Diagnostics, NY, USA) and ImmunoCAP™ (Thermo Fisher Scientific, Uppsala, Sweden) are dominant. Both platforms provide analysis of IgE to a wide range of allergens, including crude HDM and EW extracts and the HDM and EW allergen components Der p 1 and Der p 2 and Gal d 1, Gal d 2 and Gal d 4, respectively. Earlier comparisons of the two systems have revealed both significant differences in test results as well as similar performances [17–19]. The aim of this study was to compare IMMULITE and ImmunoCAP with respect to the whole extract assays of HDM and EW as well as the Der p 1, Der p 2, Gal d 1, Gal d 2 and Gal d 4 specific IgE assays.

Gradually declining levels of egg specific IgE and milder symptoms on accidental exposure or in food challenge are signs of tolerance development. At the age of 16 years, around 70% of egg allergic children will have developed tolerance [20].

## Materials and methods

Forty-four HDM-positive and 36 EW-positive serum samples were included in the study. For the EW, samples were selected from a patient pool where all patients had been diagnosed as having egg allergy while for the HDM, samples were selected from sensitized patients but with no access to their clinical diagnosis. Of each sample a series of three dilutions (1/2, 1/4, 1/8) was prepared using the ImmunoCAP IgE/ECP/Tryptase Sample Diluent. Hence, including undiluted specimens, all samples were available at four different concentrations for the assay comparisons. In a dilution linearity study, the observed (O) IgE concentration measured in each sample dilution point was evaluated against the expected (E) value derived from the measured IgE concentration in the undiluted sample, divided by the dilution factor, as an O/E ratio. For each of HDM and EW, an average O/E ratio was calculated from the O/E ratios of all dilution measurements. Weighted linear regression lines were fitted to the data with either ImmunoCAP or IMMULITE ratios as the dependent variables and ImmunoCAP / IMMULITE initial sIgE values as the independent variables.

Five allergen-specific mouse-human chimeric monoclonal IgE antibodies were also employed in the evaluation, each consisting of an allergen-specific variable domain from a mouse monoclonal antibody and the constant part of the heavy chain of human IgE. The antibodies reacted specifically with Der p 1, Der p 2, Gal d 1, Gal d 2 and Gal d 4, respectively, and could be measured with the specific and total IgE assays of both assay platforms. Furthermore, for each Ab a 6-step dilution series was prepared, up to a dilution factor of 16. Hence, samples with seven different concentrations of each Ab were available for the assay comparisons. The chimeric IgE antibodies to Der p 1, Gal d 1, Gal d 2 and Gal d 4 were obtained from Thermo Fisher Scientific (Uppsala, Sweden) while the chimeric IgE antibody against Der p 2 was obtained from Indoor Biotechnologies (Charlottesville, Virginia, US).

Competition experiments were carried out using mouse monoclonal IgG antibodies against Der p 1 and Gal d 2 (Thermo Fisher Scientific) and a mouse/human IgG4 antibody against Der p 2 (Indoor Biotechnologies). Each mouse monoclonal antibody was added at three different concentrations (0.125, 0.25 and 0.5 µg/mL) to the anti-Der p 1 or anti-Gal d 2 chimeric IgE antibody samples. Analysis was performed with specific IgE assays for HDM and EW, respectively. The results were evaluated as ratios to a control to which buffer alone had been added instead of the competing IgG antibody.

The anti-Der p 2 chimeric IgG4 antibody was added at three different concentrations (2, 20 and 200 µg/mL) to two HDM-positive serum samples (13.4 and 78.7 kU_A_/L, respectively), followed by measurement of HDM-specific IgE. The results were evaluated as ratios to a control to which buffer alone had been added. The concentration of Der p 2 specific IgG4 antibody was determined using the ImmunoCAP specific IgG4 assay (Thermo Fisher Scientific). A total of 4 comparative studies were thus performed in this work, summarized in Table 1. All ImmunoCAP measurements were performed with a Phadia 250 instrument and all IMMULITE measurements were performed with IMMULITE 2000.

**Table 1:** Summary of performed comparison studies between ImmunoCAP and IMMULITE

Comparison of:	Assays IMMULITE ImmunoCAP	Type of study	Type of comparison
Whole extract	EW (n = 36) sIgE HDM (n = 44) sIgE	Correctness by sample dilution linearity	Ratio of observed value and expected value (O/E)
Allergen-specific mouse-human chimeric IgE monoclonal antibodies	Der p 1, Der p 2 Gal d 1, Gal d 2, Gal d 4 sIgE Total IgE (ImmunoCAP)	Accuracy by sIgE/tIgE agreement in dilution series	Ratio of observed value and total IgE value (sIgE/tIgE)
Mouse monoclonal ab (IgG) addition test with 3 concentrations:		Interference study	Ratio of observed value to the control (initial level with no addition of mIgG ab)
Anti-Der p 1 mIgG	HDM sIgE		
Anti-Gal d 2 mIgG	EW sIgE		
Addition of Der p 2-chimeric IgG4 antibody to 2 sIgE positive patient samples previously tested for house dust mite (13.4 kU_A_/L and 78.7 kU_A_/L)	HDM sIgE	Interference study	Ratio of observed value to the control (initial level with no addition of IgG4 ab)

## Results

### Dilution linearity assessment with patient sera

**Fig. 1:**
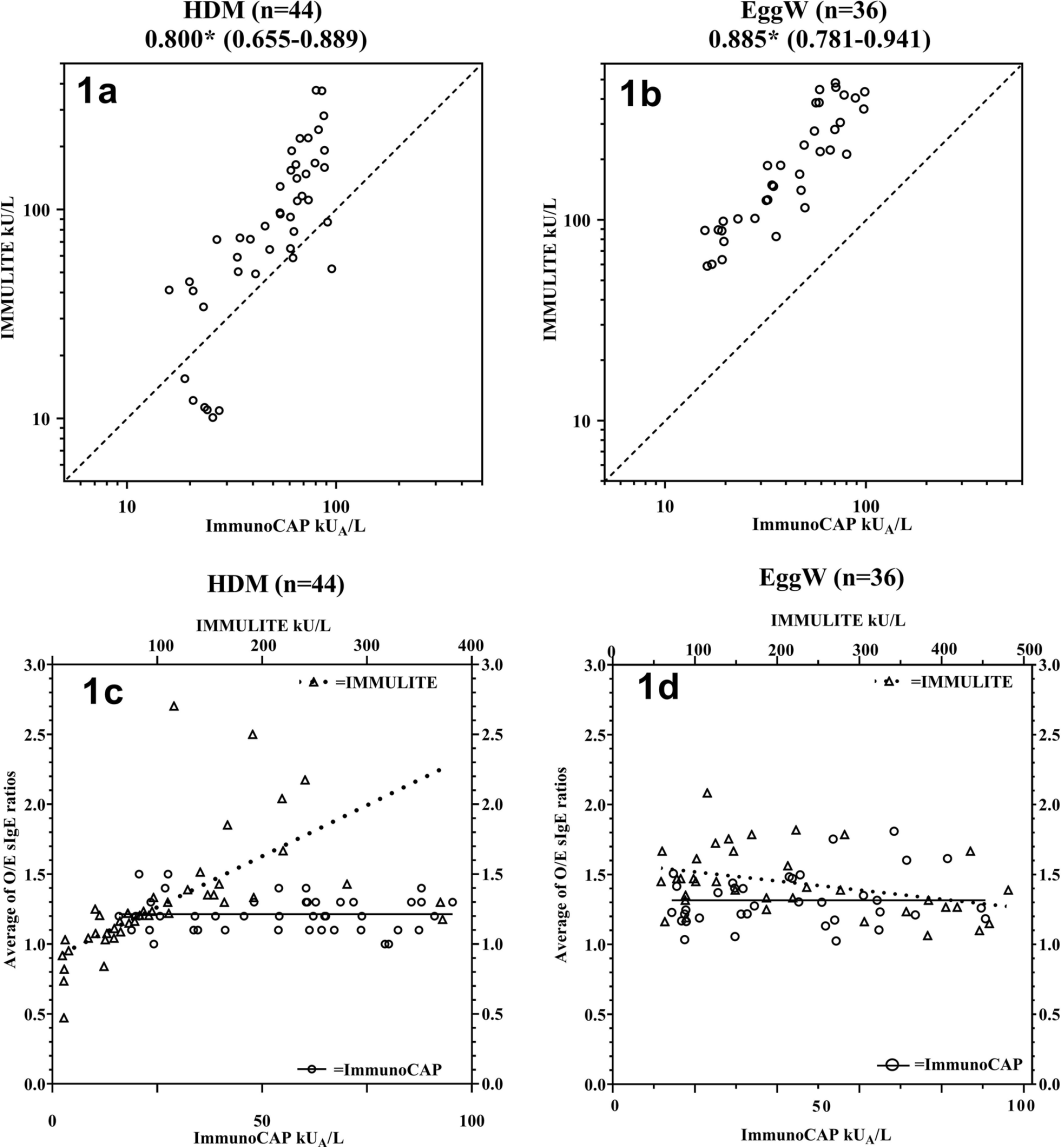
**1a** and **1b** sIgE concentration by ImmunoCAP and Immulite. with correlation and 95% CI. * P < 0.001 (P values were calculated using Spearman’s correlation coefficient). **1c **and** 1d**. Linear weighted regression (Table 2) for HDM (**1a**) and EW (**1b**) on dilution linearity with patient serum. The observed (O) results from the analyzes in each dilution series (1/2, 1/4 and 1/8) were divided by the expected (E) value derived from the measured IgE concentration in the undiluted sample. Finally, for each patient and based on the three O/E ratios, an average value was calculated. The closer to the value 1 on the Y axis, the more accurate is the test

A fundamental property required to ensure accurate measurement of an analyte by a quantitative assay is dilution linearity, i.e. that measurements returned by the assay are affected proportionally to the dilution of the analyzed specimen. To assess dilution linearity of the ImmunoCAP and IMMULITE HDM and EW IgE assays in this study, we prepared and analyzed a dilution series of 44 HDM and 36 EW positive patient samples. With ImmunoCAP, the HDM sIgE values ranged between 15.9 and 95.4 (median 60.5) and the EW sIgE values between 15.8 and 98.6 (median; 47.2) kU_A_/L. The corresponding values obtained with IMMULITE were 9.5–372 (median 88.2) kU/L for HDM and 58.9–481 (median; 47.2) kU/L for EW (Fig. 1a and 1b).

The ImmunoCAP assays displayed dilution linearity with average O/E ratios of 1.21 (range: 1–1.5; CV% = 9.8) and 1.26 (range: 1–1.8; CV% = 15.0) for HDM and EW, respectively. The IMMULITE assays displayed dilution linearity with average O/E ratios of 1.29 (range: 0.5–2.7; CV% = 32.5) and 1.44 (range: 1.1–2.1; CV% = 16.3) for HDM and EW, respectively. Weighted linear regression lines were fitted to the data as shown in Fig. 1c and d (Table 2). The closer to the value 1 on the Y axis, the more accurate is the test over its measuring range.

**Table 2:** Slope, 95% CIs,and P values for weighted regressions lines for Fig. 1

Allergen	Labarotory system	Slope	95% CI	P value
House dust mite (d1)	ImmunoCAP	− 0.0003	− 0.0019 to 0.0014	0.6789
	IMMULITE	0.0037	0.0023 to 0.0050	< 0.0001
Egg white (f1))	ImmunoCAP	0.0016	− 0.0010 to 0.0043	0.1383
	IMMULITE	− 0.0007	− 0.0012 to − 0.0001	0.0286

Compared with ImmunoCAP, IMMULITE HDM showed greater range of variation in the O/E results (p < 0.001). As demonstrated, the IMMULITE HDM assay ratios increased with increasing levels of sIgE. For both ImmunoCAP and IMMULITE EW, the variation ranges in the O/E result were quite similar while the mean value of the O/E ratio was higher for IMMULITE EW (p < 0.001).

### Specific and total IgE consistency assessment with chimeric IgE antibodies

**Fig. 2:**
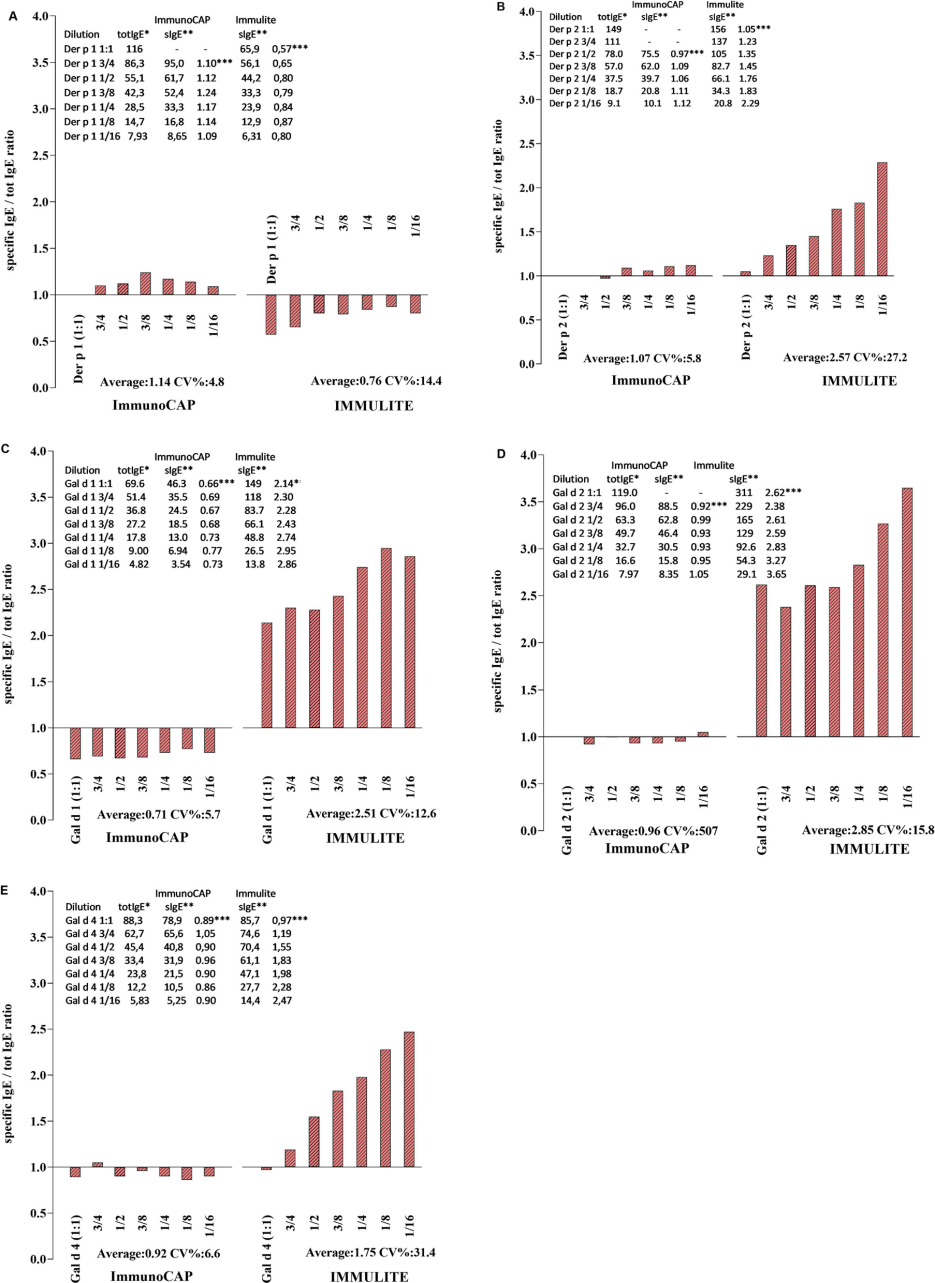
With chimeric ab (**A** Der p 1; **B** Der p 2; **C** Gal d 1, **D** Gal d 2; **E** Gal d 4) at 7 different dilution factors, specific IgE / total IgE ratio for Der p 1, Der p 2, Gal d 1, Gal d 2 and Gal d 4 allergen component. The closer to the value of 1 at the Y axis the more accurate is the test. Inset: Observed levels of specific IgE, total IgE and their ration. *kU/L; **kUA/L; ***specific IgE/total IgE

In a sample of a monoclonal IgE antibody, where all IgE is represented by a single allergen specificity, measurements of total and specific IgE should yield comparable concentration values. With ImmunoCAP, the levels of sIgE to Der p 1, Der p 2, Gal d 2 and Gal d 4 measured in all 6 dilutions of the chimeric IgE antibodies were comparable to the total IgE levels measured in the same antibody samples, with an average sIgE/tIgE ratio close to 1 (range 0.92–1.14) (Fig. 2). For the anti-Gal d 1 chimeric IgE antibody, the corresponding ratio was 0.71.

In contrast, IMMULITE gave specific IgE levels that were substantially higher than the corresponding total IgE values in all 6 dilutions of anti-Der p 2, Gal d 1, Gal d 2 and Gal d 4 chimeric IgE antibodies, with an average sIgE/tIgE ratio ranging between 1.75 and 2.85. The sIgE/tIgE ratio for the anti-Der p 1 chimeric IgE antibody was markedly lower (0.76) in IMMULITE.

### Competition experiment using monoclonal IgG and chimeric IgE antibodies

**Fig. 3:**
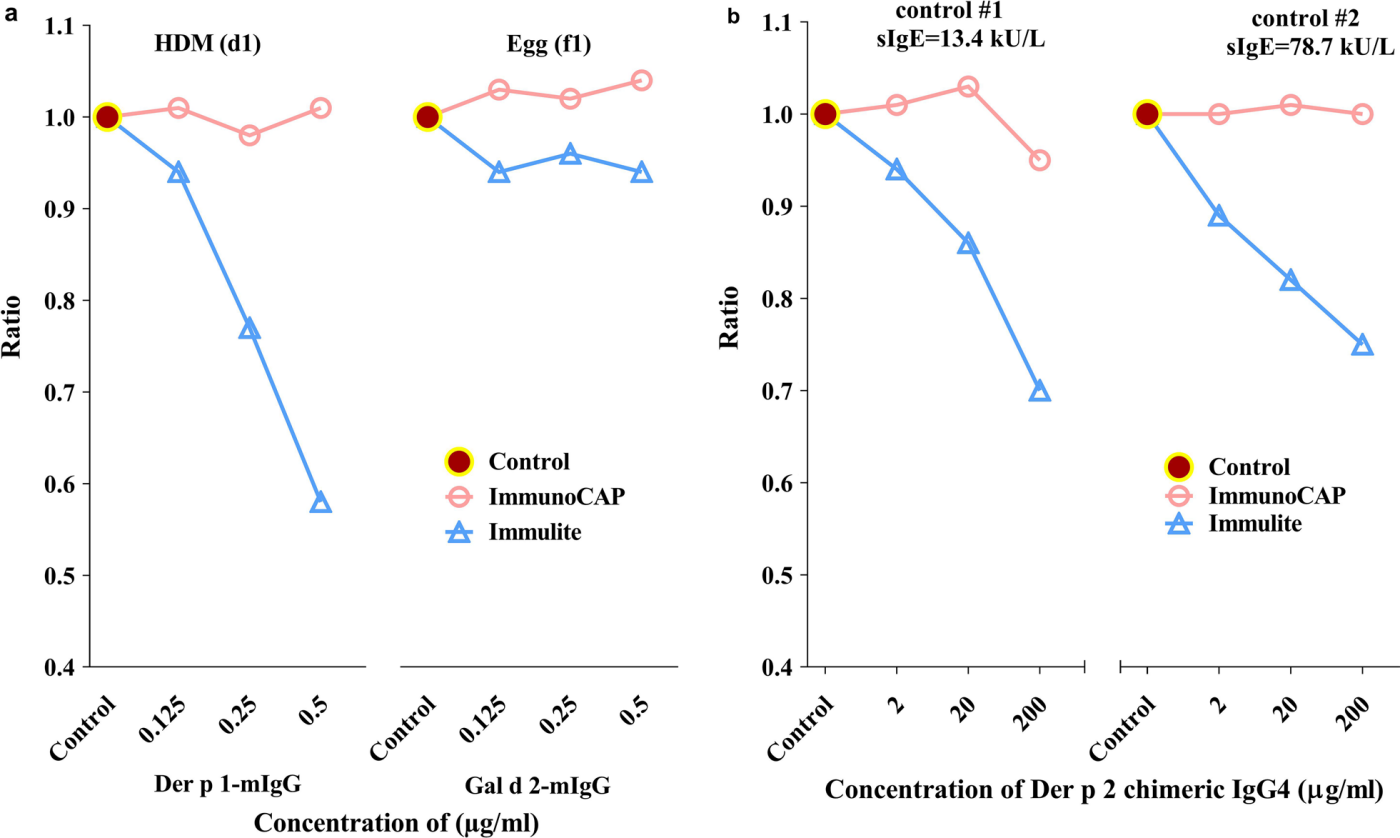
**a** Addition of anti Der p 1 mouse IgG monoclonal ab (Der p 1-mIgG) to Der p 1 chimeric IgE ab and Gal d 2 mouse IgG monoclonal ab (Gal d 2-mIgG) to Gal d 2 chimeric IgE ab at three concentrations. **b** Measurements with HDM (d1) in two control samples, #1:sIgE = 13.4 kU_A_/L; #2:sIgE = 78.7 kU_A_/L with added anti Der p 2 chimeric IgG4 at three concentrations. Results in **a** and **b** are presented as the ratio between sIgE in each of the treated samples and the untreated control

A robust assay is characterized by its ability to deliver consistent results from specimens regardless of their content of potential interferents. One important cause of potential interference is allergen specific non-IgE antibodies which may be induced by natural allergen exposure or allergen immunotherapy (AIT) treatment. In this study, we assessed the resilience of the ImmunoCAP and IMMULITE HDM and EW IgE assays against such interference by competing with the increasing concentrations of monoclonal IgG antibodies of the same allergen specificity prior to IgE measurement. The results of this experiment are shown in Fig. 3a. Addition of anti-Der p 1 or anti-Gal d 2 mouse monoclonal IgG antibodies exerted no significant blocking effect on the measurement of the corresponding chimeric IgE antibodies with the ImmunoCAP HDM and EW IgE assays. Also, with IMMULITE, measurement of the anti-Gal d 2 chimeric IgE antibody with the EW assay was largely unaffected by the addition of the anti-Gal d 2 mouse monoclonal IgG antibody. However, addition of increasing concentrations of the anti-Der p 1 mouse monoclonal IgG antibody substantially outcompeted the anti-Der p 1 chimeric IgE antibody in the IMMULITE HDM IgE assay, causing the measured IgE values to drop by up to 42%.

### Competition experiment using monoclonal IgG4 and patient sera

While the above competition experiment using chimeric IgE antibodies addressed the IgE binding capacity of the assays in an analytical set-up, we also studied their ability to withstand competing antibodies in a situation more closely reflecting their clinical use. This was done by assessing the effect on HDM IgE measurements in patient samples by increasing concentrations of a chimeric Der p 2 specific IgG4 antibody. The results of this experiment are shown in Fig. 3b. Addition of the anti-Der p 2 chimeric IgG4 antibody to either of the two patient sera studied caused no change in IgE antibody concentration measured with the ImmunoCAP HDM IgE assay. In contrast, with the IMMULITE HDM IgE assay, the measured IgE antibody concentration decreased substantially with increasing amount of added chimeric IgG4, by up to 30 and 25% in the two sera, respectively.

## Discussion

In this study, the ability of the ImmunoCAP and IMMULITE IgE assay systems to accurately quantify allergen specific IgE antibodies was studied. Main findings were generally poor agreement between the two systems in the measurement of specific IgE levels to HDM and EW. This is especially true for the results in the linearity study where the effect in IMMULITE HDM of the serial dilutions on sIgE recovery was more variable compared to ImmunoCAP. Furthermore, for IMMULITE EW, mean sIgE results from the diluted samples was higher than for the results with ImmunoCAP (1.44 vs 1.29). In a perfect linearity dilution study, the mean O/E ratio should be 1.

Differences in assay performance between the two systems were further investigated using chimeric IgE antibodies. In a sample where all IgE antibodies are represented by a single allergen specificity, measurements of total and specific IgE should yield comparable concentration values. Since a close agreement of total IgE concentrations measured with the IMMULITE and ImmunoCAP systems has earlier been demonstrated [21] and both are calibrated against the current WHO reference preparation of IgE, substantially divergent specific and total IgE results can only be attributed to the specific IgE assays. For 4 of the 5 chimeric IgE antibodies analyzed in the study (Der p 2, Gal d 1, Gal d 2 and Gal d 4), it was found that the IMMULITE system substantially overestimated the amount of specific IgE while the concentration of the chimeric anti-Der p 1 IgE antibody was somewhat underestimated. In contrast, the concentrations of 4 of the 5 chimeric IgE antibodies (Der p 1, Der p 2, Gal d 2 and Gal d 4) were more accurately determined in the ImmunoCAP system, with specific IgE/total IgE ratios close to 1, while the chimeric anti-Gal d 1 IgE antibody was somewhat underestimated.

The two interference studies performed showed that IgE measurements with the ImmunoCAP HDM and EW IgE assays were unaffected by the addition of the competing IgG or IgG4 antibodies, as was the IMMULITE EW IgE assay. In contrast, significant interference was observed with the IMMULITE HDM IgE assay, both when the chimeric anti-Der p 1 IgE antibody and human patient samples were measured in competition with anti-Der p 1 IgG and anti-Der p 2 IgG4, respectively, with a reduction of measured specific IgE values by up to 42%. The important implication of the observed interference is that levels of IgE to HDM will not be accurately measured in patients who have mounted an IgG response against HDM, such as during HDM immunotherapy.

Taken together, this study demonstrates significant differences in performance of specific IgE assays between the two assay systems with respect to quantitative accuracy. Additionally, different resistance to interference by competing IgG antibodies was demonstrated which may affect IgE measurement in patients treated with allergen immunotherapy. The findings highlight that despite the use of the same concentration units of specific IgE and calibration against the WHO reference preparation of IgE, different test results for a patient sample may be obtained depending on the assay system used by the laboratory. As a consequence, different diagnostic conclusions may be drawn, affecting the management of the patient.

A limitation in this study is the relatively small number of sera in the dilution linearity part, with 44 and 36 sera for the HDM and EW group, respectively. A study with a greater number of sera, spread evenly over the entire measuring range, may have provided for a more robust and representative comparison of the assay systems.

## Conclusions

This study demonstrates significant differences in analytical performance of two specific IgE assay systems for whole extracts and individual allergen components. The results indicate higher accuracy of quantitation of specific IgE and a higher resistance to competing allergen-specific non-IgE antibodies of ImmunoCAP as compared to IMMULITE.

## Data Availability

The authors confirm that all data underlying the findings are fully available without restriction. All relevant data are within the paper.

## References

[1] Ulrik CS, Backer V (1999). Markers of impaired growth of pulmonary function in children and adolescents. Am J Respir Crit Care Med.

[2] Bousquet PJ, Chinn S, Janson C, Kogevinas M, Burney P, Jarvis D (2007). Geographical variation in the prevalence of positive skin tests to environmental aeroallergens in the European Community Respiratory Health Survey I. Allergy.

[3] Chew GL, Reardon AM, Correa JC, Young M, Acosta L, Mellins R, Chew FT, Perzanowski MS (2009). Mite sensitization among Latina women in New York, where dust-mite allergen levels are typically low. Indoor Air.

[4] Wang JY, Chen WY (1992). Inhalant allergens in asthmatic children in Taiwan: comparison evaluation of skin testing, radioallergosorbent test and multiple allergosorbent chemiluminescent assay for specific IgE. J Formos Med Assoc.

[5] Calderon MA, Linneberg A, Kleine-Tebbe J, De Blay F, Hernandez Fernandez de Rojas D, Virchow JC, Demoly P (2015). Respiratory allergy caused by house dust mites: what do we really know?. J Allergy Clin Immunol.

[6] Becker S, Rasp J, Eder K, Berghaus A, Kramer MF, Groger M (2016). Non-allergic rhinitis with eosinophilia syndrome is not associated with local production of specific IgE in nasal mucosa. Eur Arch Otorhinolaryngol.

[7] Banerjee S, Weber M, Blatt K, Swoboda I, Focke-Tejkl M, Valent P, Valenta R, Vrtala S (2014). Conversion of Der p 23, a new major house dust mite allergen, into a hypoallergenic vaccine. J Immunol.

[8] Rona RJ, Keil T, Summers C, Gislason D, Zuidmeer L, Sodergren E, Sigurdardottir ST, Lindner T, Goldhahn K, Dahlstrom J, McBride D, Madsen C (2007). The prevalence of food allergy: a meta-analysis. J Allergy Clin Immunol.

[9] Benhamou AH, Caubet JC, Eigenmann PA, Nowak-Wegrzyn A, Marcos CP, Reche M, Urisu A (2010). State of the art and new horizons in the diagnosis and management of egg allergy. Allergy.

[10] Komata T, Soderstrom L, Borres MP, Tachimoto H, Ebisawa M (2007). The predictive relationship of food-specific serum IgE concentrations to challenge outcomes for egg and milk varies by patient age. J Allergy Clin Immunol.

[11] Sampson HA, Ho DG (1997). Relationship between food-specific IgE concentrations and the risk of positive food challenges in children and adolescents. J Allergy Clin Immunol.

[12] Sato S, Ogura K, Takahashi K, Sato Y, Yanagida N, Ebisawa M (2017). Usefulness of antigen-specific IgE probability curves derived from the 3gAllergy assay in diagnosing egg, cow's milk, and wheat allergies. Allergol Int.

[13] Eigenmann PA (2000). Anaphylactic reactions to raw eggs after negative challenges with cooked eggs. J Allergy Clin Immunol.

[14] Leonard SA, Sampson HA, Sicherer SH, Noone S, Moshier EL, Godbold J, Nowak-Wegrzyn A (2012). Dietary baked egg accelerates resolution of egg allergy in children. J Allergy Clin Immunol.

[15] Palosuo K, Kukkonen AK, Pelkonen AS, Makela MJ (2018). Gal d 1-specific IgE predicts allergy to heated egg in Finnish children. Pediatr Allergy Immunol.

[16] Ando H, Moverare R, Kondo Y, Tsuge I, Tanaka A, Borres MP, Urisu A (2008). Utility of ovomucoid-specific IgE concentrations in predicting symptomatic egg allergy. J Allergy Clin Immunol.

[17] Al Hawi Y, Nagao M, Furuya K, Sato Y, Ito S, Hori H, Hirayama M, Fujisawa T (2021). Agreement between predictive, allergen-specific IgE values assessed by ImmunoCAP and IMMULITE 2000 3gAllergy^TM^ assay systems for milk and wheat allergies. Allergy Asthma Immunol Res.

[18] Park KH, Lee J, Sim DW, Lee SC (2018). Comparison of singleplex specific IgE detection immunoassays: ImmunoCAP Phadia 250 and Immulite 2000 3gAllergy. Ann Lab Med.

[19] Szecsi PB, Stender S (2013). Comparison of immunoglobulin E measurements on IMMULITE and ImmunoCAP in samples consisting of allergen-specific mouse-human chimeric monoclonal antibodies towards allergen extracts and four recombinant allergens. Int Arch Allergy Immunol.

[20] Savage JH, Matsui EC, Skripak JM, Wood RA (2007). The natural history of egg allergy. J Allergy Clin Immunol.

[21] Wood RA, Segall N, Ahlstedt S, Williams PB (2007). Accuracy of IgE antibody laboratory results. Ann Allergy Asthma Immunol.

